# Environmental effects on the lung and gastrointestinal parasite fauna of wild boar: a comparative study between alpine and Mediterranean ecosystems

**DOI:** 10.1007/s11686-026-01309-x

**Published:** 2026-06-20

**Authors:** Camilla Sangiovanni, Valerio Orazi, Irene Belardi, Leonardo Gallotta, Francesco Ferretti, Sonia Calderola, Patrizia Malaspina, Isabel Guadano-Procesi, Carlotta Fiorilla, David Di Cave, Federica Berrilli

**Affiliations:** 1 Department of Clinical Sciences and Translational Medicine, Tor Vergata University of Rome, Rome, Italy; 2 Ecology and Environmental Health, Tor Vergata University of Rome, Rome, Italy; 3 Department of Life Sciences, University of Siena, Siena, Italy; 4 PNRR (NBFC - National Biodiversity Future Center, Palermo, Italy; 5 Parco Nazionale Gran Paradiso, Turin, , Italy; 6 Department of Biology, Tor Vergata University of Rome, Rome, Italy; 7 PhD Courses in Microbiology, Immunology, Infectious Diseases and Transplants (MIMIT), Faculty of Medicine and Surgery, Tor Vergata University of Rome, Rome, Italy

**Keywords:** Wild boar, Helminths, Protozoa, Elevation, Habitat

## Abstract

**Purpose:**

This study aims to explore environmental factors associated with parasite infections in faecal samples collected from wild boar populations, by analysing the prevalence, intensity, and abundance of parasite communities across two Italian ecosystems: the alpine environment in the Orco Valley (Gran Paradiso National Park) and the Mediterranean lowland in the Maremma Regional Park.

**Methods:**

Seasonal faecal samples were collected from November 2023 to July 2025 along systematic transects in both study areas. Samples were analysed using the Mini-FLOTAC technique to quantify parasite eggs and (oo)cysts, and prevalence, intensity, and abundance were calculated. Environmental variables, including elevation, land use, temperature, precipitation, and season, were linked to each sample. Generalized linear models (GLMs) were fitted to evaluate the effects of environmental factors on parasite presence and abundance.

**Results:**

Parasite communities were dominated by *Eimeria* spp., gastrointestinal strongyles, and *Metastrongylus* spp., whereas other taxa (e.g., *Balantioides coli*, *Capillaria* spp., *Cystoisospora* sp.) were sporadic. Significant differences in intensity and/or abundance of dominant taxa were observed between the two areas. Models based on infection abundance, rather than presence, revealed environmental associations: *Metastrongylus* spp. abundance decreased with increasing elevation, possibly reflecting constraints on intermediate host availability; while gastrointestinal strongyles abundance was lower in open natural habitats, reflecting microclimatic influences on larval survival.

**Conclusions:**

This study highlights associations between habitat characteristics and parasite community structure in wild boar populations, providing insights into the environmental factors potentially influencing parasite transmission across heterogeneous landscapes.

**Supplementary Information:**

The online version contains supplementary material available at 10.1007/s11686-026-01309-x.

## Introduction

The wild boar *Sus scrofa* is a worldwide distributed large mammal, occupying a wide range of natural, agricultural, and peri-urban habitats across Europe [1]. After being intensively hunted and driven to local extinction in many areas of the continent by the end of the nineteenth century, populations have since undergone a rapid increase in both abundance and distribution [2]. Its increasing population density and expanding geographic range have intensified the interactions with domestic pigs, livestock, companion animals, and humans, often generating ecological, economic, and social conflicts and public health risks [3].

As a generalist and highly adaptable species, the wild boar plays a major role in European ecosystems, and it is of increasing relevance from both ecological and management perspectives [4]. Its foraging behaviour, particularly rooting for invertebrates and below-ground plant material, can profoundly modify soil structure, vegetation composition, and habitat characteristics [4, 5].

Parasites represent a major component of biodiversity and play a fundamental role in shaping ecological and evolutionary processes. They act as powerful evolutionary factors, influencing host genetic and phenotypic diversity, the expression of secondary sexual traits, and the structure of biological communities [6, 7]. By affecting host body condition, reducing reproductive success, and increasing mortality, parasites can significantly modify population dynamics and alter the topology of food webs, including chain length, connectance, and robustness. Variations in parasite community composition have therefore been widely used as bioindicators of environmental stress, biodiversity loss, climatic conditions, and shifts in host age structure [8]. Owing to their often complex life cycles, parasites can integrate long-term information on host feeding ecology, thereby providing valuable insights into trophic interactions within ecosystems and revealing ontogenetic changes in host diet [9].

Parasite community composition and structure in wild boar are closely associated with ecological features. Environmental factors, such as vegetation cover, soil characteristics, moisture levels, temperature regimes, and land-use patterns, can influence the survival, development, and dispersal of parasite stages. For example, forested areas with dense understory and high humidity may promote the persistence of protozoan cysts, oocysts, and nematode eggs. Likewise, rooting activity would be expected to enhance contact with contaminated soil layers. Moreover, access to water bodies or muddy wallows may facilitate the transmission of environmentally resistant stages. Environmental variables may also affect the distribution and abundance of intermediate hosts involved in indirect parasite life cycles [10].

Lung and gastrointestinal parasites of wild boar differ substantially in their life cycles, transmission routes, and environmental requirements. Some taxa are transmitted directly through environmental stages, while others depend on intermediate hosts. These differences suggest that parasite taxa may respond differently to habitat features, but the relative importance of such factors in natural wild boar remains poorly understood. Despite the ecological and public health relevance of wild boar, information on how different environmental variables contribute to parasite communities across different landscapes remains limited [11].

In this context, the present study aims to explore potential environmental factors that may influence the composition and structure of lung and gastrointestinal parasite communities in wild boar. For this purpose, the study considered two natural protected areas of Central and Northern Italy, including a Mediterranean coastal habitat dominated by sclerophyllous scrubwood and a mountainous landscape. These areas represent markedly different environmental settings, allowing the exploration of parasite–environment associations across distinct ecological contexts. Environmental predictors were selected because of their known or potential relevance to parasite transmission processes, either through effects on free-living stages, resistant environmental forms, intermediate hosts, or host habitat use.

Understanding how habitat features contribute to structure parasite assemblages is crucial for interpreting infection patterns in wild boar and for assessing their role as reservoirs of pathogens of veterinary and public health importance. Therefore, wild boar could represent a model system for exploring how environmental gradients, and host behaviour interact to structure lung and gastrointestinal parasite communities in protected natural landscapes.

## Materials and methods

### Study areas

#### Alpine ecosystem

**Fig. 1:**
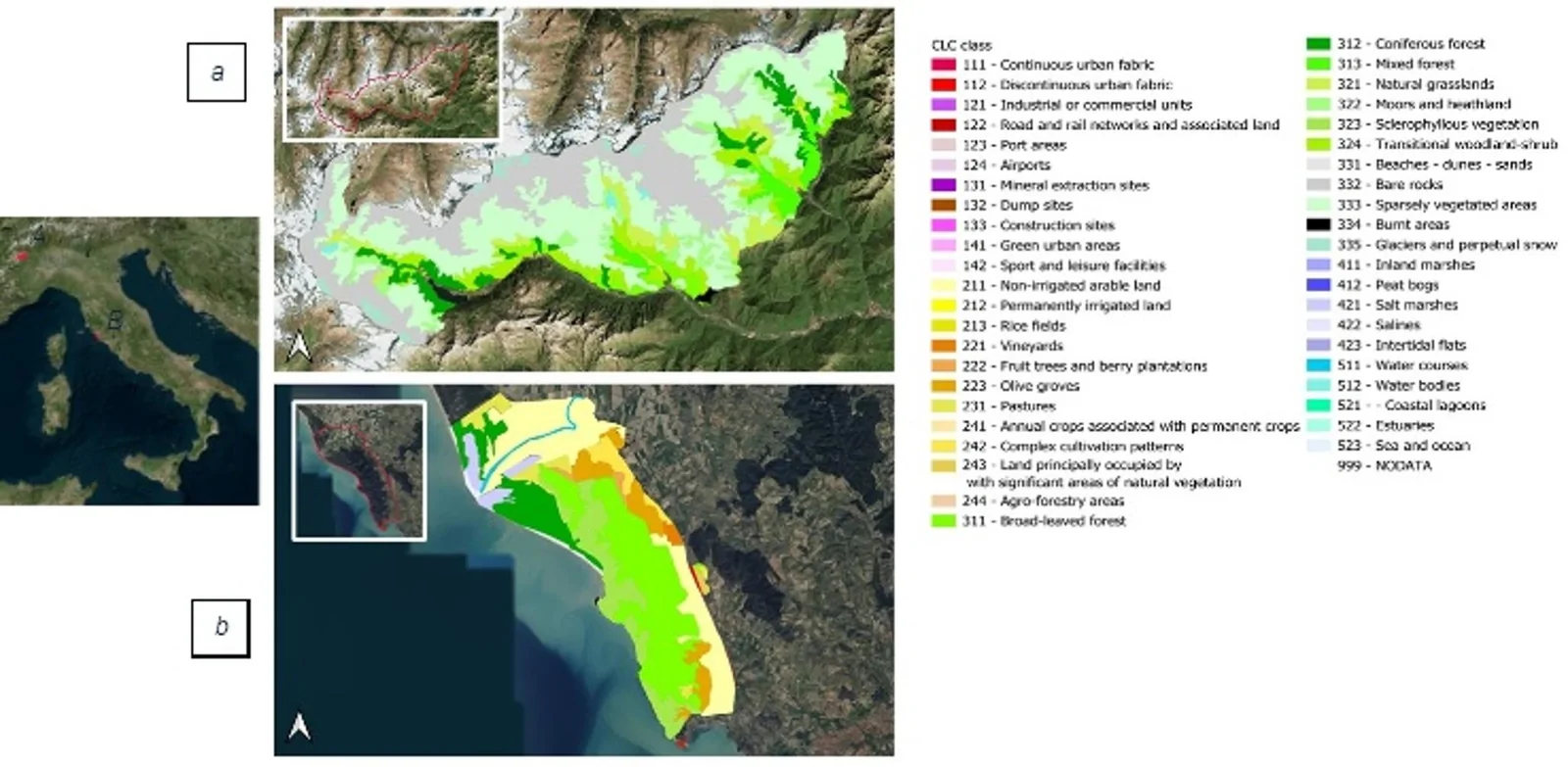
Map of study areas. Red lines indicate the boundaries of the **a** Gran Paradiso National Park and **b** Maremma Regional Park. The maps show the distribution of CORINE Land Cover classes (CLC, 2018); each colour represents a distinct land cover category according to the CLC classification. Coordinates are in the WGS 84 Reference System. Maps created using the Free and Open Source QGIS 3.22.0 ‘Białowie˙za’ (https://www.qgis.org)

The Orco Valley extends for approximately 40 km on the south side of the Gran Paradiso National Park (GPNP), in the Western Italian Alps (45°09’ N, 7°51' E). The valley ranges from ~ 600 m to over 3,000 m a.s.l., a broad elevational gradient typical of alpine ecosystems. However, the sampling sites included in this study were restricted to a narrower elevational range (674–1223 m a.s.l.). The climate is alpine continental, characterized by long winters and persistent snow covers, which strongly influence habitat accessibility, animal movements, and the environmental persistence of parasite resistant stages [12]. Vegetation follows a typical alpine zonation, with mixed conifer forests and pastures at lower elevations (600–1,500 m), subalpine larch stands and montane meadows between 1,600 and 2,200 m, and alpine grasslands and scree slopes above the tree line (~ 2,200–2,400 m) [13]. In addition to the presence of wild boar, the valley supports populations of Alpine ibex *Capra ibex*, Alpine chamois *Rupicapra rupicapra rupicapra*, and, locally, red deer *Cervus elaphus* and roe deer *Capreolus capreolus*. These species move seasonally along the altitudinal gradient, creating spatial and temporal variability in faecal deposition patterns and exposure of parasite stages to environmental conditions. Wild boar began recolonizing the study area in the 1980s [14] and currently occurs at relatively low densities, estimated at 2.1 individuals per km^2^ based on camera-trapping surveys (Panaccio et al. unpublished results). Seasonal grazing by domestic livestock (cattle, sheep, and goats) occurs in alpine grasslands during summer months [15], while human disturbance remains limited, particularly within the park’s core protection zones [16]. The study area is shown in Fig. 1.

#### Mediterranean ecosystem

The Maremma Regional Park (MRP) is located in Central Italy (42°39′ N, 11°05′ E) and covers approximately 90 km^2^, extending from the Tyrrhenian coast to the inland, with elevations up to 417 m a.s.l. The climate is Mediterranean, characterised by hot, dry summers and mild, rainy winters, generating a markedly seasonal context for parasite development and transmission. The landscape is dominated by Mediterranean sclerophyllous vegetation. Forest and shrub communities are mainly composed of *Quercus ilex*, *Juniperus* spp., *Myrtus communis*, *Phillyrea* spp., *Cistus* spp., *Arbutus unedo*, *Pistacia lentiscus,* and *Salvia rosmarinus*. In the northern part of the park, a large domestic pine (*Pinus pinea* L.) forest is present. Wild ungulate species include also the roe deer, and the fallow deer *Dama dama*. Wild boar densities, estimated from faecal counts carried out in summer, were relatively stable over the study period, averaging between 11 and 13 individuals per km^2^ [17]. Livestock is also present, including sheep, cattle, and horses, with a combined density of about 20 individuals per km^2^. Cattle and horses graze freely in several areas, and calving generally occurs during the winter months [17]. Human settlements occupy less than 2% of the total surface [17, 18].

The study area is reported in Fig. 1.

### Faecal samples collection

Faecal samples were collected seasonally from November 2023 to July 2025 along predefined transects and sectors established to ensure systematic coverage of the two study areas. The search was conducted during the early morning to maximise detectability of fresh droppings and minimise degradation due to diurnal temperature increases. The search targeted exclusively wild boar faecal pellets, identified through their characteristic size and morphology. Only fresh and recently deposited samples, as identified by moisture, colour, and integrity, were collected to reduce biases related to environmental contamination and deterioration of parasite stages. To reduce the likelihood of repeated sampling from the same individual, only faecal samples with a minimum distance of 20 m were collected along transects, this spatial criterion helps to increase independence among samples. The collected material was placed in a sterile container and labelled with the sample ID, date, and GPS coordinates. Samples were transported to the laboratory under refrigerated conditions, stored at 4 °C, and processed promptly, usually within 24 h.

### Coproparasitological analysis

Faecal samples were analysed using the Mini-FLOTAC technique [19]. The flotation media consisted of either saturated zinc sulfate (ZnSO₄; specific gravity = 1.350) or saturated sodium chloride (NaCl; specific gravity = 1.200) and both flotation solution were used systematically for all samples. Briefly, two grams of fresh faeces for each sample were placed into the Fill-FLOTAC container and diluted with 18 mL of flotation solution, resulting in a 1:10 faeces-to-solution ratio. From each flotation solution, 2 mL of suspension was added to two Mini-FLOTAC chambers and left to float for 10 min. Oocysts, cysts, eggs and larval forms were identified by morphometric analysis under a light microscope (Olympus CX40). Faecal egg and (oo)cyst counts were calculated by multiplying the total number of eggs or (oo)cysts observed in both chambers by five, yielding eggs or (oo)cysts per gram of faeces, reported as EPG and (O)CPG, respectively. Parasite prevalence, mean abundance, and mean intensity were calculated according to Bush et al. [20] using quantitative eggs or (oo)cysts counts. Confidence intervals for prevalence were estimated using the Wilson method.

### Environmental data collection

Environmental variables, including climatic and habitat-related predictors, were selected based on their known or expected influence on parasite survival and development (e.g. sporulation or egg embryonation rates) and on host exposure to infective stages. Geographic coordinates (X, Y) were used to extract environmental data for each sampling point. Elevation was obtained from the AWS digital elevation model at 12 m resolution using the elevatr R package and was included as a proxy for broad-scale variation in temperature, radiation, and seasonality. Land cover was assigned using the 2018 Corine Land Cover (CLC) dataset. Sampling points were spatially joined to the CLC polygons to obtain CLC codes and classes. Original CLC classes were aggregated into three major categories (CLCgroup) to reduce model complexity: Forest (broad-leaved forest, mixed forest, sclerophyllous vegetation), Open Natural Habitats (natural grasslands, pastures), and Agricultural Areas (non-irrigated arable land, complex cultivation patterns). Reclassification was performed in R using the dplyr package. Land cover was included to capture habitat-specific microclimatic conditions and differences in host use of the landscape, both of which may affect parasite persistence and transmission.

Temperature, precipitation and solar radiation were extracted from WorldClim 2.1 climate layers at 2.5 arc-minute resolution, monthly values correspond to long-term average climatic conditions. Mean monthly temperature (°C) and total monthly precipitation (mm) were obtained for each point, while solar radiation (MJ/m^2^/day) represents the mean daily incoming energy for the corresponding month. Climatic variables were included because they directly influence free-living parasite stages. Coordinates were transformed to WGS84, and monthly raster layers were used. In addition, the date of collection was used to assign the samples to a season. All environmental variables were merged with the main dataset, ensuring their association with each faecal sample.

### Data analysis

Differences between the two parks were assessed using Fisher’s exact test for prevalence, and the Wilcoxon rank-sum test for infection intensity and abundance. Statistical significance was evaluated based on p-values. The influence of environmental variables on parasite presence and abundance was assessed using generalized linear models. The models were fitted using the dominant parasite taxa as response variables. Parasite occurrence (presence/absence) was analysed using binomial generalized linear models (GLM), with study area, elevation, habitat categories (CLCgroup, with three levels: Forest, Open Natural Habitats and Agricultural Areas), mean monthly temperature in °C, mean monthly precipitation in mm and season (winter: December–February, spring: March–May, summer: June–August, and autumn: September–November) as predictors. Parasite abundance was analysed using negative binomial GLM with the same predictors. Solar irradiation was initially considered as a predictor but was excluded from the final models due to high collinearity with other environmental variables, as indicated by variance inflation factors’ (VIF) values. A threshold of VIF > 3 was used to indicate problematic collinearity [21]. Inference was based on a global model including ecologically relevant predictors a priori, in order to evaluate the conditional contribution of each variable while accounting for the effects of the others. Significance was assessed at p < 0.05. All data processing, visualization, and statistical analyses were conducted in R version 4.4.3 using the following packages: dplyr, tidyr, ggplot2, vegan, FactoMineR, factoextra, openxlsx, glmmTMB, performance, DHARMa, car, lme4, MASS, and broom. Residual diagnostics were performed using the *DHARMa* package in R with 1,000 simulations per model [22].

## Results

### Parasitological results

**Fig. 2:**
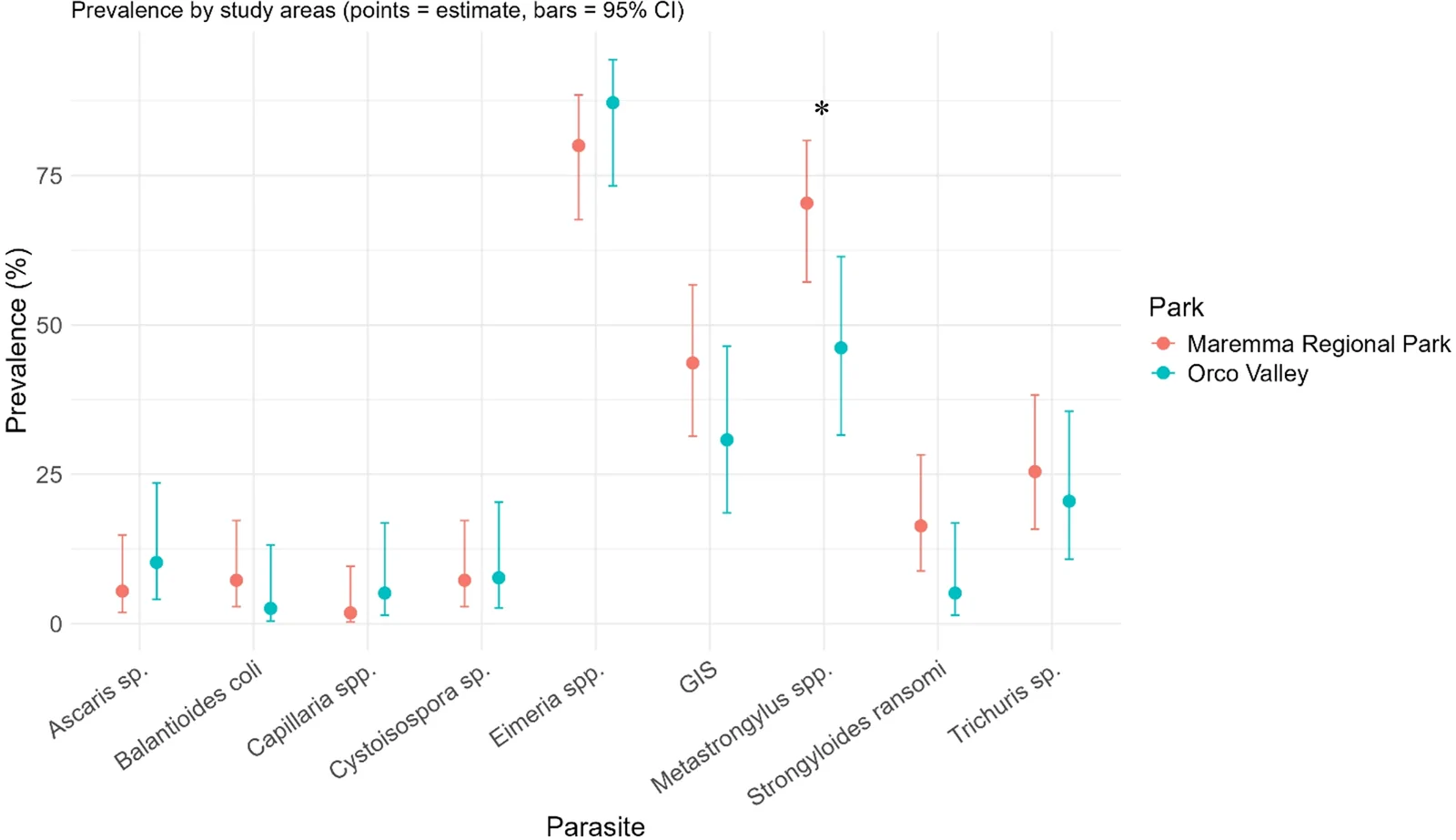
Dot plot showing parasite prevalence in the two parks, with bars representing 95% confidence intervals. Significance levels are indicated with asterisks: * p < 0.05

A total of 94 wild boar faecal samples were analysed from the two study areas: Orco Valley (n = 39) and Maremma Regional Park (n = 55). Parasites were detected in most samples, with only two samples from Orco Valley and three from Maremma Regional Park showing no detectable parasites. Nine parasite taxa were detected from both areas (Table 1 and Fig. 2). *Eimeria* spp. was the most prevalent parasite, detected in 87.2% (95% CI: 73.3–94.4%) of samples in the Orco Valley and 80.0% (95% CI: 67.6–88.4%) in MRP. *Cystoisospora* sp. and *Balantioides coli* occurred at low prevalence in both areas, ranging from 2.6% to 7.7%. Helminth eggs identified as *Ascaris* sp., *Capillaria* spp., gastrointestinal strongyles (GIS)*, Strongyloide*s *ransomi*, and *Trichuris* sp. were also detected, with prevalence varying across the two areas. Notably, *Metastrongylus* spp. were significantly more prevalent in Maremma Regional Park (69.1%, 95% CI: 55.9–79.7%) than in the Orco Valley (46.1%, 95% CI: 31.6–61.4%; p < 0.05).

**Table 1:** Parasite prevalence in wild boar faecal samples from two parks. Number of positives (%) for each parasite, with 95% confidence intervals

	Orco Valley (n = 39)		Maremma regional park (n = 55)		
Taxa	Positive (%)	95%–CI	Positive (%)	95%—CI	p-values
*Eimeria* spp.	34 (87.2)	73.3–94.4	44(80)	67.6–88.4	0.526
*Cystoisospora* sp.	3 (7.69)	2.65–20.3	4 (7.27)	2.86–17.3	1.000
*Balantioides coli*	1 (2.56)	0.454–13.2	4 (7.27)	2.86–17.3	0.399
*Ascaris* sp.	4 (10.3)	4.06–23.06	3 (5.45)	1.87–14.9	0.444
*Capillaria* spp.	2 (5.13)	1.42–16.9	1 (1.82)	0.32–9.61	0.568
GIS	12 (30.8)	18.6–46.4	24 (43.6)	31.4–56.7	0.294
*Metastrongylus* spp.	18 (46.1)	31.6–61.4	38 (69.09)	55.9–79.7	0.043 *
*Strongyloides ransomi*	2 (5.13)	1.42–16.9	9 (16.4)	8.86–28.3	0.115
*Trichuris* sp.	8 (20.5)	10.8–35.5	14 (25.5)	15.8–38.3	0.756

Significance levels are indicated with asterisks: * p< 0.05

The highest intensity of protozoan infection in the Orco Valley was observed for *Balantioides coli* (195 CPG), although this result was obtained from a single positive sample, followed by *Eimeria* spp. (93.97 OPG), while in MRP is shown by *Eimeria* spp. (379.4 OPG), followed by *Balantioides coli* (80 CPG). Regarding metazoans, in both areas, *S. ransomi* showed the highest intensity of infection (100 EPG in the Orco Valley and 125 EPG in MRP) (Table 2). As for abundance, Eimeria spp. reached the highest values in both the Orco Valley and MRP (81.92 OPG and 303.54 OPG, respectively). Regarding helminths, Metastrongylus spp. were the most abundant (17.94 EPG) taxa in the Orco Valley, whereas in MRP were GIS with 42.36 EPG (Table 2 ).

**Table 2:** Parasite infection intensity and abundance. Mean, median, and range of infection intensity (eggs or (oo)cysts per gram) and mean abundance for each parasite taxon in the two parks

Taxa	Orco Valley (n = 39)				Maremma Regional Park (n = 55)					
	I		A		I		A		pI	pA
	Mean	Median	Range	Mean	Mean	Median	Range	Mean		
*Eimeria* spp.	93.97	55	5–335	81.92	379.4	277.5	10–1720	303.54	*** < 0.001	**0.007
*Cystoisospora* sp.	20	10	10–40	1.54	25	22.5	5–50	1.81	-	0.946
*Balantioides coli*	195 ᵟ	/	/	5	80	50	5–215	5.82	0.8	0.334
*Ascaris* sp.	23.33	30	10–85	3.97	23.33	20	10–40	1.27	0.716	0.376
*Capillaria* spp.	22.5	22.5	20–25	1.15	35^ᵟ^	/	/	0.64	0.667	0.392
GIS	19.17	5	5 -110	5.89	97.08	67.5	5–330	42.36	**0.002	*0.045
*Metastrongylus* spp.	38.90	27.5	5 -120	17.94	36.71	25	5–125	25.36	0.832	*0.04
*Strongyloides ransomi*	100	100	100—100	5.13	125	75	25–375	20.45	0.905	0.102
*Trichuris* sp.	21.87	20	5–55	4.48	26.43	22.5	5–60	6.72	0.704	0.545

I = intensity of infection; A = abundance of infection; p-values for intensity (pI) and abundance (pA) of the parasites detected in the samples. Significance levels are indicated with asterisks: * p < 0.05; ** p < 0.01; *** p < 0.001

^δ^This value is referred to cysts or eggs observed in just one faecal sample

**Table 3:** Effects of study area and ecological variables on parasite occurrence (binomial generalized linear models)

Parasite	Predictor	Estimate (logit)	SE	Z	p value
*Eimeria* spp.	(Intercept)	-3.412	5.391	-0.633	0.527
	Park [Gran Paradiso *vs.* Maremma]	-2.826	4.716	-0.599	0.549
	CLC_group [Forest *vs.* Agicultural areas]	-0.316	1.669	-0.189	0.850
	CLC_group [Open_natural *vs.* Agicultural areas]	-0.553	1.660	-0.333	0.739
	Season [Spring *vs.* winter]	4.524	4.228	1.070	0.285
	Season [Summer *vs.* winter]	6.956	7.509	0.926	0.354
	Season [Autumn *vs.* winter]	-1.844	2.569	-0.718	0.473
	Temp month °C	-0.088	0.477	-0.185	0.853
	Prec (mm)	0.085	0.059	1.448	0.148
	Elevation (m)	0.002	0.003	0.667	0.505
*Metastrongylus* spp.	(Intercept)	1.621	2.972	0.545	0.585
	Park [Gran Paradiso *vs.* Maremma]	2.086	2.344	0.890	0.374
	CLC_group [Forest *vs.* Agicultural areas]	-0.395	0.909	-0.435	0.663
	CLC_group [Open_natural *vs.* Agicultural areas]	0.800	1.035	0.773	0.440
	Season [Spring *vs.* winter]	0.704	1.353	0.520	0.603
	Season [Summer *vs.* winter]	0.265	2.226	0.119	0.905
	Season [Autumn *vs.* winter]	0.941	1.449	0.649	0.516
	Temp month °C	-0.040	0.179	-0.224	0.823
	Prec (mm)	-0.015	0.032	-0.469	0.639
	Elevation (m)	-0.003	0.002	-1.282	0.200
GIS	(Intercept)	-2.114	3.011	-0.702	0.482
	Park [Gran Paradiso *vs.* Maremma]	-1.120	2.532	-0.442	0.658
	CLC_group [Forest *vs.* Agicultural areas]	-0.264	0.918	-0.288	0.773
	CLC_group [Open_natural *vs.* Agicultural areas]	-1.249	1.015	-1.231	0.218
	Season [Spring *vs.* winter]	-0.146	1.526	-0.096	0.924
	Season [Summer *vs.* winter]	0.688	2.615	0.263	0.793
	Season [Autumn *vs.* winter]	-1.172	1.655	-0.708	0.479
	Temp month °C	0.024	0.200	0.121	0.904
	Prec (mm)	0.046	0.034	1.355	0.175
	Elevation (m)	-0.001	0.002	-0.513	0.608

Reference levels are the *Maremma Regional Park* for the Park and *Agricultural land cover* for the CLC group, *winter* for the Season. Estimates are shown on the logit scale

The results of the statistical tests on parasite intensity and abundance between the two study areas are summarised in Table 2. Significant differences were detected in the intensity of infection for *Eimeria* spp. (***p < 0.001) and GIS (***p < 0.002), and in the abundance of infection for *Eimeria* spp. (***p < 0.007), GIS (*p < 0.045) and *Metastrongylus* spp. (*p < 0.04).

**Fig. 3:**
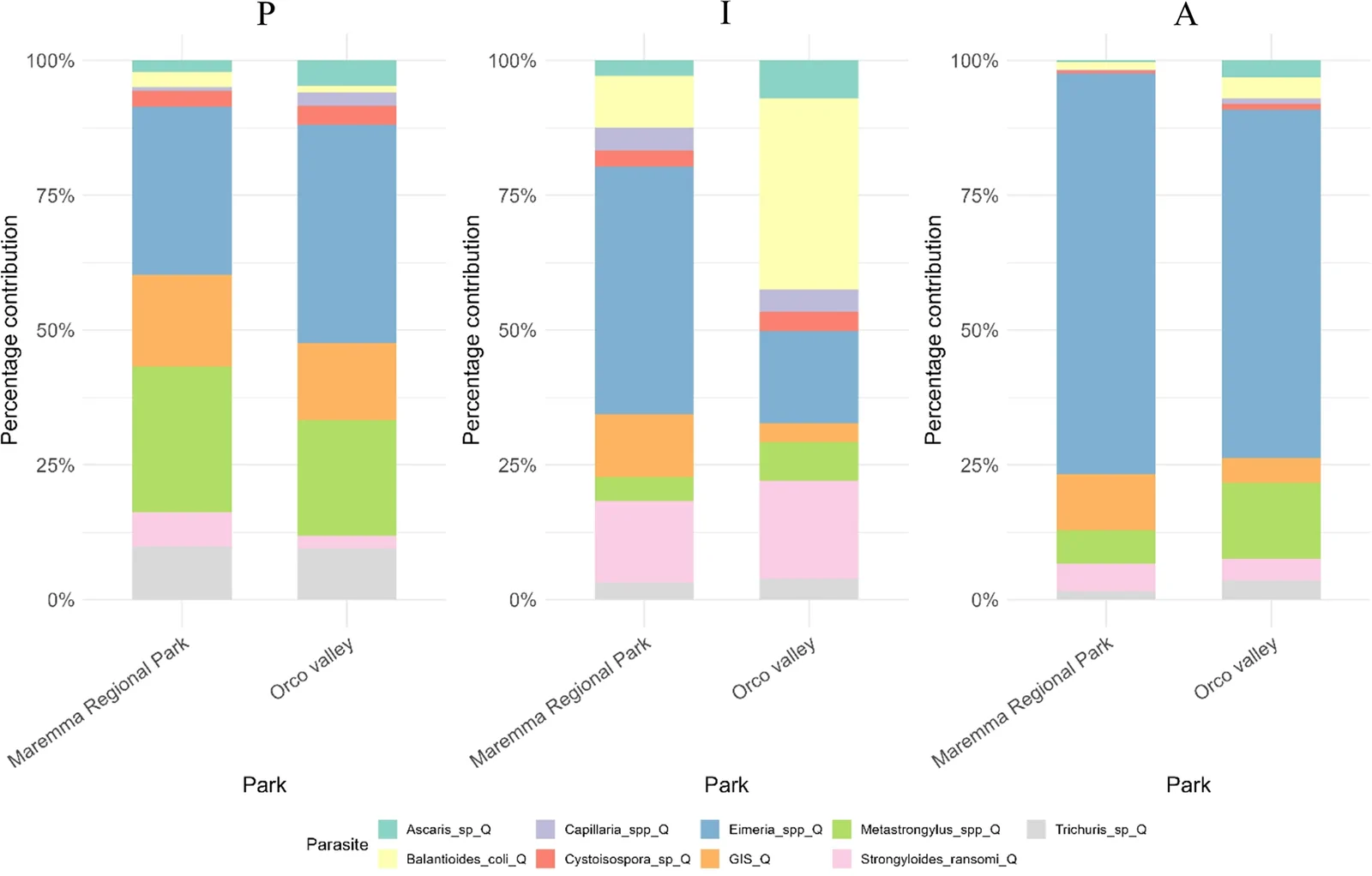
Barplots showing the relative contribution of each parasite taxon to the (P) prevalence, (I) mean intensity, and (A) mean abundance of infection in wild boar faecal samples from Maremma Regional Park and Gran Paradiso National Park (Orco Valley)

The relative contribution of each parasite taxon to the overall values of prevalence, infection intensity, and abundance is shown in Fig. 3.

### Models’ results

Environmental characteristics of the sampling sites were assessed for each study area and summarised in Table S1 of Supplementary materials. In the Orco Valley, sampling locations ranged from 674 to 1223 m in elevation, with monthly temperatures between -1.49 and 4.84 °C, and precipitation from 73 to 101 mm. In contrast, Maremma Regional Park (MRP) sampling sites were located at lower elevations from 0 to 146 m, higher temperatures from 7.9 to 25.6 °C, and precipitation from 19 to 88 mm.

The modelling analyses focused on the three most prevalent and abundant taxa detected in both parasite communities: *Eimeria* spp., *Metastrongylu*s spp., and gastrointestinal strongyles. These three parasites were selected as response variables in the modelling analyses.

As shown in Table 3, the GLMs fitted to parasite occurrence did not reveal significant effects of park identity or environmental variables for any of the taxa investigated. The probability of infection for *Eimeria* spp., *Metastrongylus* spp., and gastrointestinal strongyles were not significantly influenced by park, habitat category, season, temperature, precipitation, or elevation.

In contrast, the GLMs fitted on parasite abundance (Table 4) revealed more structured patterns, showing significant associations with specific environmental predictors depending on taxon. For *Metastrongylus* spp., elevation emerged as a significant predictor of abundance, with higher elevations associated with reduced parasite loads. For gastrointestinal strongyles the abundance was significantly influenced by habitat categories. In particular, Open Natural Habitats were associated with markedly lower parasite abundance compared to other habitat categories.

For *Eimeria* spp., no environmental variables was statistically significant, indicating no detectable association with variation in oocyst counts.

Model diagnostics for all fitted models, reported in Figure S1 – S6 of the supplementary materials.

**Table 4:** Effects of park identity and ecological variables on parasite abundance (negative binomial generalized linear models)

Parasite	Predictor	Estimate (log)	SE	z	pvalue
*Eimeria* spp.	(Intercept)	3.576	2.224	1.608	0.108
	Park [Gran Paradiso *vs.* Maremma]	-1.859	1.801	-1.032	0.302
	CLC_group [Forest *vs.* Agicultural areas]	0.030	0.693	0.044	0.965
	CLC_group [Open natural *vs.* Agicultural areas]	-0.366	0.745	-0.491	0.623
	Season [Spring *vs.* winter]	0.210	1.010	0.208	0.835
	Season [Summer *vs.* winter]	0.489	1.688	0.290	0.772
	Season [Autumn *vs.* winter]	-0.970	1.124	-0.863	0.388
	Temp_month	0.052	0.135	0.384	0.701
	Prec_mm	0.031	0.024	1.270	0.204
	Elevation	0.000	0.002	0.177	0.859
*Metastrongylus* spp.	(Intercept)	3.576	2.599	1.376	0.169
	Park [Gran Paradiso *vs.* Maremma]	3.258	2.103	1.549	0.121
	CLC_group [Forest *vs.* Agicultural areas]	-0.066	0.808	-0.081	0.935
	CLC_group [Open natural *vs.* Agicultural areas]	0.232	0.868	0.267	0.790
	Season [Spring *vs.* winter]	0.235	1.188	0.198	0.843
	Season [Summer *vs.* winter]	0.088	1.983	0.044	0.965
	Season [Autumn *vs.* winter]	-0.456	1.340	-0.341	0.733
	Temp month °C	-0.042	0.159	-0.262	0.793
	Prec (mm)	0.010	0.029	0.337	0.736
	Elevation (m)	-0.005	0.002	-3.020	0.003**
GIS	(Intercept)	7.117	4.288	1.660	0.097
	Park [Gran Paradiso *vs.* Maremma]	-13.043	3.838	-3.398	0.001
	CLC_group [Forest *vs.* Agicultural areas]	-0.626	1.345	-0.465	0.642
	CLC_group [Open natural *vs.* Agicultural areas]	-3.262	1.476	-2.209	0.027*
	Season [Spring *vs.* winter]	2.970	2.374	1.251	0.211
	Season [Summer *vs.* winter]	5.573	4.270	1.305	0.192
	Season [Autumn *vs.* winter]	0.378	2.149	0.176	0.860
	Temp month °C	-0.439	0.313	-1.405	0.160
	Prec (mm)	0.032	0.046	0.699	0.485
	Elevation (m)	0.005	0.003	1.741	0.082

Estimates are shown on the log scale and represent changes in mean parasite load. Reference levels are the *Maremma Regional Park* for the Park and *Agricultural land cover* for the CLC group, *winter* for the Season. Significant effects (*p < 0.05; ** *p* < 0.01) are shown in bold

## Discussion

This study provides a comparative assessment of lung and gastrointestinal parasites detected in wild boar faecal samples collected from two ecologically different environments in Italy—an alpine ecosystem (Gran Paradiso National Park, Orco Valley) and a Mediterranean lowland ecosystem (Maremma Regional Park, MRP). Compared to the Orco Valley, MRP represents a multi-host system in which wild and domestic ungulates share habitats within a highly heterogeneous Mediterranean landscape throughout the year. In contrast, in the Alpine area spatial overlap between wild and domestic ungulates is expected to occur mainly during the warmer periods, particularly in summer. These conditions provide a contrasting ecological context for coproparasitological investigations. By integrating coproparasitological data with environmental variables, results offer insights into how some habitat characteristics and ecological variables may influence parasite occurrence, and abundance in a generalist wild ungulate, such as the wild boar.

Overall, parasite community composition was relatively homogeneous between the two study areas, with the same nine taxa detected in both. Some taxa, including *Trichuris* sp., *Ascaris* sp. and *S. ransomi,* are commonly found in Italian wild boar [23, 24]. *Capillaria* spp. infections in wild boar are rarely documented and generally occur at low prevalence [23]; recently Pacifico et al. [25] reported the first identification of *Eucoleus garfiai* (syn. *Capillaria garfiai*) in wild boars from Southern Italy. To date, there are no records of *Balantioides coli* or *Cystoisospora* sp. in Italy, the latter being reported at low prevalence in Poland [26].

The three taxa dominating the parasite communities were *Eimeria* spp., *Metastrongylus* spp., and gastrointestinal strongyles (GIS). *Eimeria* spp. was the most prevalent taxon, in agreement with previous studies across Europe where these coccidia were always present, generally common, and often abundant in wild boar populations [27, 28]. Reported prevalences range from 47.5% in urban–suburban populations in Poland to as high as 92.3% in Danish populations [11, 26]. Lower prevalence values were recorded in the only available Italian study on wild boar coccidians conducted in the Umbria region, where *Eimeria* spp. were detected at comparatively reduced frequencies [23]. However, *Eimeria* spp. exhibited the highest values of both intensity and abundance in both study areas, particularly in Maremma Regional Park, in line with numerous coproparasitological surveys identifying coccidia as the most quantitatively dominant protozoan parasites in wild boar [23]. The high prevalence and abundance of *Eimeria* spp. likely results from a combination of the parasites' ecological traits and host–environment interactions: *Eimeria* spp. oocysts are highly resistant to environmental conditions, allowing them to persist in soil and other substrates for extended periods while remaining infective. This persistence facilitates the accumulation of infective stages in areas frequently used by wild boar, such as rooting sites, thereby enhancing transmission opportunities. In addition, the high degree of host specificity and long-term coevolution with suids promote efficient transmission and maintenance of *Eimeria* spp. populations across wild and domestic hosts [27, 28]. It is important to underline that infections of *Eimeria* spp . in *Sus scrofa* are generally subclinical in adult animals and rarely cause overt disease under natural conditions, with clinical coccidiosis being mainly associated with young or immunocompromised individuals [29].

*Metastrongylus* spp., the only parasite with a dixenous life cycle detected in this study, was also a dominant taxon, with prevalence, abundance and intensity in agreement with previous reports from both Italy and other parts of Europe [22, 26, 30, 31]. Notably, prevalence and abundance were significantly higher in the Maremma regional park compared to the Orco Valley, reflecting local environmental conditions that may be compatible with the development of the intermediate hosts, the earthworms, as evidenced in the abundance model.

Finally, gastrointestinal strongyles (GIS), showed high prevalence in both study areas, consistent with previously reported ranges for European wild boar populations and reflecting the widespread distribution of this group of nematodes [30]. Regarding abundance and intensity, significant differences were observed between the Maremma Regional Park and the Orco Valley, with higher values recorded in the MRP, suggesting that local environmental conditions may influence infection levels for this broadly distributed parasite group.

Modelling analyses focused on the three dominant taxa (*Eimeria* spp., *Metastrongylus* spp. and GIS) as response variables, allowed to investigate the potential environmental variables likely influencing parasite presence and abundance. Models based on presence–absence data did not reveal significant associations with the environmental variables considered, suggesting that occurrence alone may be insufficient to detect ecological constraints on parasite transmission. The absence of significant effects in the occurrence models may be partly related to the relatively high prevalence of the dominant parasite taxa across both study areas, which likely limits variability in presence–absence data and reduces the ability of the GLMs to detect environmental associations.

On the other hand, quantitative models revealed environmental effects for *Metastrongylus* spp. and GIS. *Metastrongylus* spp. showed a negative relationship between infection abundance and elevation, suggesting that altitude may be an environmental correlate of lungworm abundance. This result is consistent with the ecology of *Metastrongylus* spp., whose indirect life cycle depends on earthworms as intermediate hosts that are highly sensitive to temperature, soil moisture, and seasonal climatic stability. Cooler temperatures, prolonged snow cover and reduced soil biological activity at higher elevations likely limit the survival and availability of intermediate hosts, thereby reducing transmission efficiency and parasite abundance. Indeed, earthworm communities decline sharply with altitude, and they may be completely absent above certain elevations, such as the alpine level [32]. Similar altitudinal or habitat-related differences in *Metastrongylus* spp. prevalence have been reported in other European studies, suggesting that lungworm infections are particularly sensitive to environmental gradients and host–parasite interactions [31, 33, 34].

Gastrointestinal strongyles (GIS) abundance showed a negative relationship with Open Natural Habitats. This result could suggest that open habitats may be less favourable for the persistence of free-living infective stages due to increased exposure to desiccation, temperature fluctuations and ultraviolet radiation, as well as potentially reduced host aggregation. Consistent conclusions have been reported for gastrointestinal nematodes in wild boar populations in Poland, where forested habitats were associated with higher humidity and greater thermal stability, promoting larval survival in the soil [34].

Collectively, these findings indicate that assessing parasite occurrence alone provides an incomplete view of infection ecology in wild boar. While presence–absence patterns were relatively uniform across contrasting environments, such as Alpine and Mediterranean ecosystems analysed in this study, quantitative metrics revealed spatial and environmental heterogenity.

From an ecological perspective, these results highlight the importance of integrating host–parasite–environment interactions into studies of wildlife disease dynamics. Parasite networks in multi-host systems, such as Maremma Regional Park, illustrate how habitat heterogeneity, interspecific interactions, and landscape structure can modulate infection patterns, providing a framework to understand the ecological aspects associated with parasite diversity and transmission. Finally, these findings have practical implications for wildlife management and disease control, quantitative assessments of infection should complement prevalence surveys to identify transmission hotspots and target interventions. Habitat management, monitoring of intermediate host populations and mitigation of overlap between wild and domestic ungulates may reduce parasite transmission risks. Collectively, combining quantitative parasitological data with environmental and ecological analyses improves our understanding of host–parasite dynamics and supports informed conservation, disease management, and One Health strategies in wild ungulate populations.

## Supplementary Information

Below is the link to the electronic supplementary material.

Supplementary Material 1

## Data Availability

No datasets were generated or analysed during the current study.
