# Supplementary material for: Environmental effects on the lung and gastrointestinal parasite fauna of wild boar: a comparative study between alpine and Mediterranean ecosystems
Source: Acta Parasitol. 2026 Jun 20;71(4):144. doi: 10.1007/s11686-026-01309-x (PMC13283150; doi:10.1007/s11686-026-01309-x)
Supplement: Supplementary file 1 — Supplementary Material 1 [file 11686_2026_1309_MOESM1_ESM.docx]

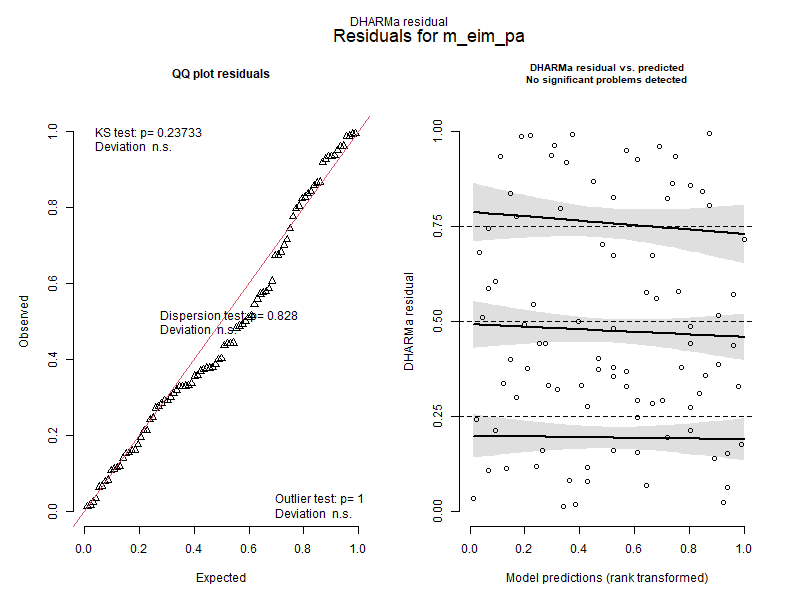


Figure S1. Diagnostic plots for the binomial model predicting the presence/absence of *Eimeria* spp.


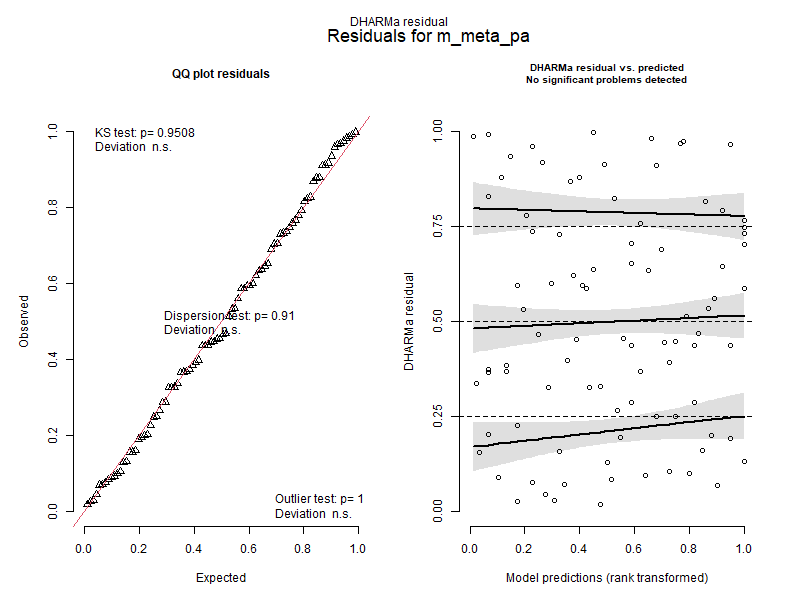


Figure S2. Diagnostic plots for the binomial model predicting the presence/absence of Metastrongylus spp.


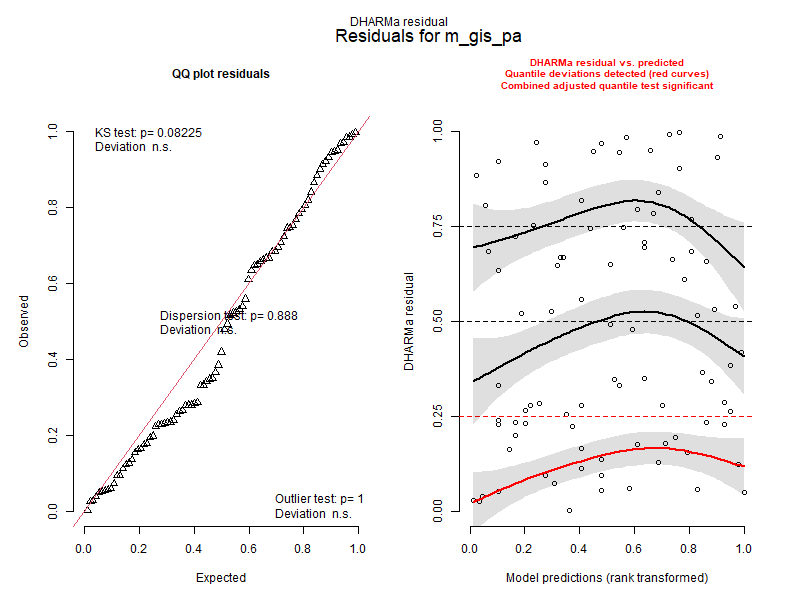


Figure S3. Diagnostic plots for the binomial model predicting the presence/absence of GIS.


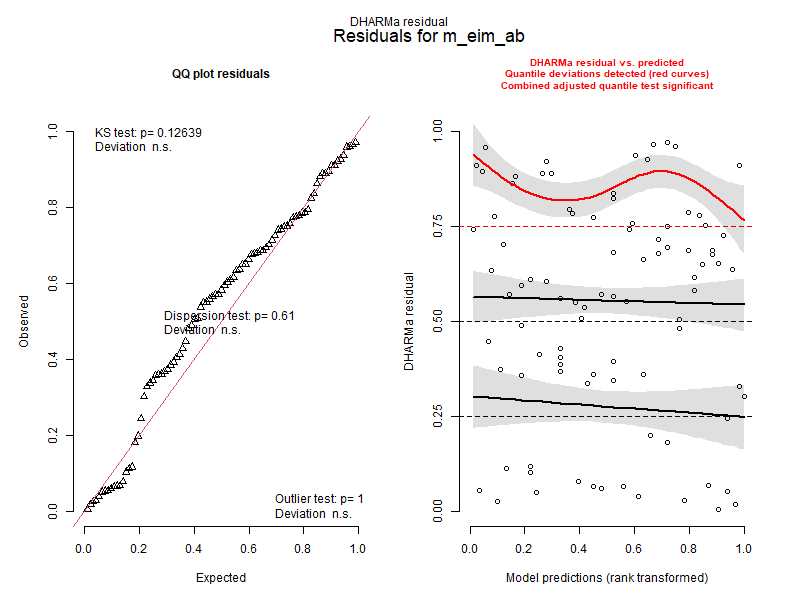


Figure S4. Diagnostic plots for the model predicting Eimeria spp. abundance.


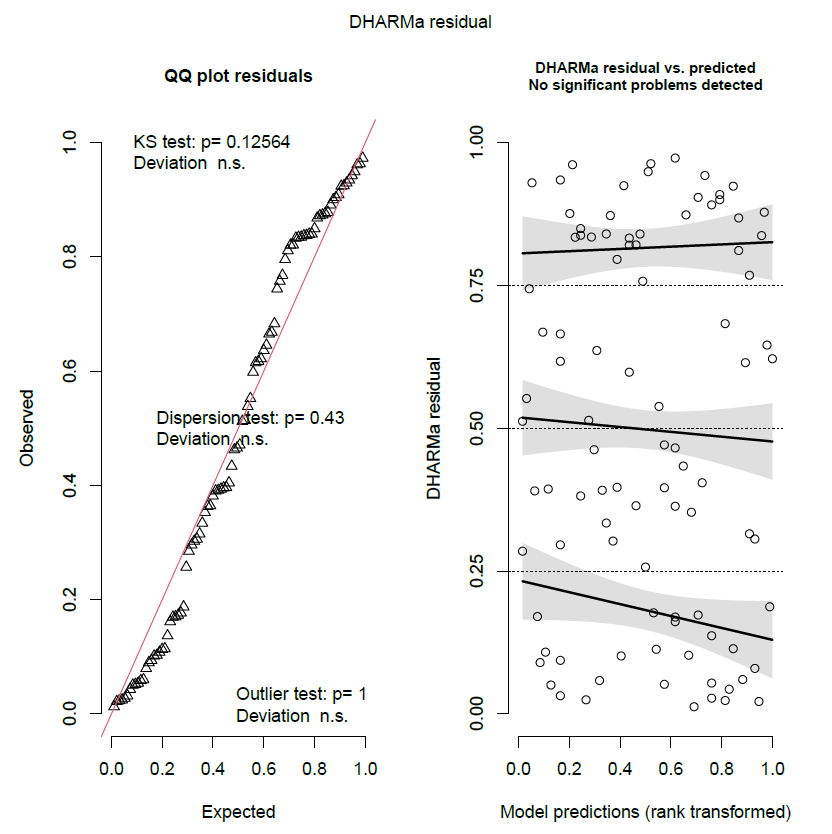


Figure S5. Diagnostic plots for the model predicting Metastrongylus spp. abundance.


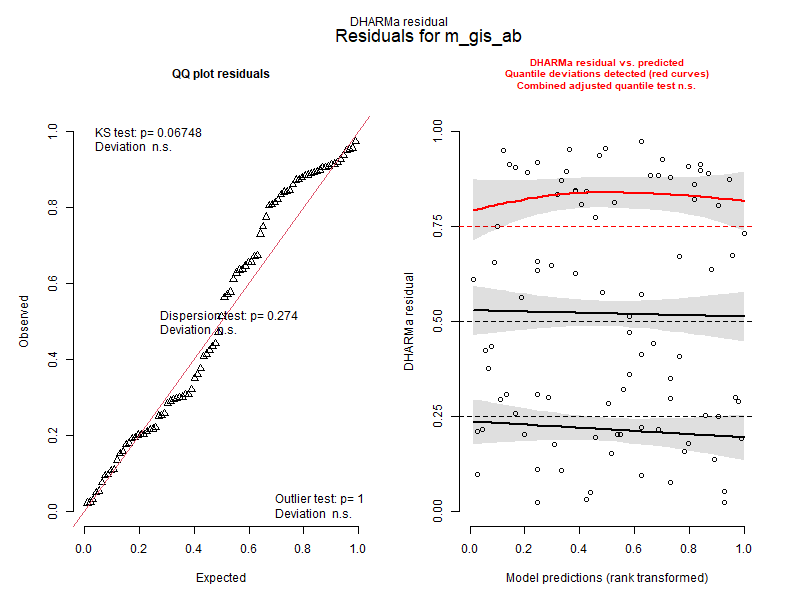


Figure S6. Diagnostic plots for the model predicting GIS abundance*.*
